# Supplementary material for: Performance evaluation of Baermann techniques: The quest for developing a microscopy reference standard for the diagnosis of Strongyloides stercoralis
Source: PLoS Negl Trop Dis. 2021 Feb 18;15(2):e0009076. doi: 10.1371/journal.pntd.0009076 (PMC7891789; doi:10.1371/journal.pntd.0009076)
Supplement: S1 Table — (DOCX) [file pntd.0009076.s002.docx]

| Diagnostic method | Item | Unit cost (local) | Unit cost ($US) | Estimated life expectancy (Year) | Estimated days per year | Cost per sample ($US) |
| --- | --- | --- | --- | --- | --- | --- |
| CB | Centrifuge | 60,000 | 1898.73 | 10 | 50 | 0.16 |
|  | Forceps | 100 | 3.16 | 2 | 20 | 0.08 |
|  | Mohr clamp | 90 | 2.85 | 2 | 20 | 0.07 |
|  | Funnel | 90 | 2.85 |  |  | 0.02 |
|  | Funnel stand | 2000 | 63.29 | 5 | 20 | 1.58 |
|  | Strainer | 50 | 1.58 |  |  | 0.02 |
|  | Petri dish | 184 | 5.82 |  |  | 0.06 |
|  | Rubber tube | 15 | 0.47 |  |  | 0.07 |
|  | Falcon tube (15mL) | 800 | 25.32 |  |  | 0.25 |
|  | Tissue paper | 300 | 9.49 |  |  | 0.18 |
|  | Activated charcoal | 1840 | 58.23 |  |  | 0.58 |
|  | Field coordinator | 300 | 9.49 |  |  | 0.4 |
|  | Field worker | 300 | 9.49 |  |  | 0.4 |
|  | Laboratory technician | 644 | 20.38 |  |  | 0.85 |
|  | Driver | 300 | 9.49 |  |  | 0.4 |
|  | Cleaner | 100 | 3.16 |  |  | 0.13 |
|  | Vehicle | 1000 | 31.65 |  |  | 1.32 |
|  | Fuel | 24 | 0.76 |  |  | 0.03 |
| MB | Falcon tube (50 mL) | 500 | 15.82 |  |  | 0.32 |
|  | Gauze | 354 | 11.20 |  |  | 0.11 |
|  | Field coordinator | 300 | 9.49 |  |  | 0.24 |
|  | Field worker | 300 | 9.49 |  |  | 0.24 |
|  | Laboratory technician | 644 | 20.38 |  |  | 0.51 |
|  | Driver | 300 | 9.49 |  |  | 0.24 |
|  | Cleaner | 100 | 3.16 |  |  | 0.08 |
|  | Vehicle | 1000 | 31.65 |  |  | 0.79 |
|  | Fuel | 24 | 0.76 |  |  | 0.02 |
| MBCI | Falcon tube (50 mL) | 500 | 15.82 |  |  | 0.32 |
|  | Gauze | 354 | 11.20 |  |  | 0.11 |
|  | Activated charcoal | 1840 | 58.23 |  |  | 0.29 |
|  | Petri dish | 184 | 5.82 |  |  | 0.06 |
|  | Field coordinator | 300 | 9.49 |  |  | 0.24 |
|  | Field worker | 300 | 9.49 |  |  | 0.24 |
|  | Laboratory technician | 644 | 20.38 |  |  | 0.51 |
|  | Driver | 300 | 9.49 |  |  | 0.24 |
|  | Cleaner | 100 | 3.16 |  |  | 0.08 |
|  | Vehicle | 1000 | 31.65 |  |  | 0.79 |
|  | Fuel | 24 | 0.76 |  |  | 0.02 |
| CB, MB, MBCI | Stool Collection cup | 7 | 0.22 |  |  | 0.22 |
|  | Toilet paper | 30 | 0.95 |  |  | 0.02 |
|  | Wooden spatulas | 136 | 4.30 |  |  | <0.001 |
|  | Disposable gloves | 120 | 3.80 |  |  | 0.08 |
|  | Permanent marker | 40 | 1.27 |  |  | 0.01 |
|  | Wooden applicator stick | 286 | 9.05 |  |  | 0.09 |
|  | Microscope slides | 95 | 3.01 |  |  | 0.3 |
|  | Cover slide | 50 | 1.58 |  |  | 0.079 |
|  | Pasture pipette | 2400 | 75.95 |  |  | 0.08 |
|  | Disposable gloves | 120 | 3.80 |  |  | 0.08 |
|  | Markers | 40 | 1.27 |  |  | 1.27 |
|  | Plastic jar | 30 | 0.95 |  |  | 0.95 |
|  | Bleach | 21 | 0.66 |  |  | 0.022 |
|  | Liquid detergent | 16 | 0.51 |  |  | 0.02 |
|  | Bucket | 70 | 2.22 |  |  | 2.22 |
|  | Brushes | 20 | 0.63 |  |  | 0.63 |
|  | Cleaners Glove | 50 | 1.58 |  |  | 1.58 |
|  | Biohazard bag | 20 | 0.63 |  |  | 0.02 |
|  | Pen | 5 | 0.16 |  |  | 0.16 |
|  | Recording sheets | 200 | 6.33 |  |  | 0.16 |
|  | Microscope | 90,000 | 2848.10 | 10 | 100 | 0.12 |
|  | Analytical scale | 57,000 | 1803.80 | 10 | 50 | 0.15 |
|  | Water Bath | 21,000 | 664.56 | 10 | 50 | 0.06 |
