# Supplementary material for: Performance evaluation of Baermann techniques: The quest for developing a microscopy reference standard for the diagnosis of Strongyloides stercoralis
Source: PLoS Negl Trop Dis. 2021 Feb 18;15(2):e0009076. doi: 10.1371/journal.pntd.0009076 (PMC7891789; doi:10.1371/journal.pntd.0009076)
Supplement: S1 Text — (DOCX) [file pntd.0009076.s003.docx]

**Standard Operating Procedure for Conventional Bearmann (CB)**

# Introduction

The Baermann technique is used to separate larvae from faecal material based on the active migration or movement of larvae downward from fresh stool samples to water of warmer temperature. Faeces are suspended in water. After permitting sufficient time to allow migration, the supernatant of the warm water will be discarded and the sediment will be examined microscopically for the presence of the larvae.

# Safety

- Stool samples should be treated as potentially infectious and universal precautions should be always followed
- Avoid contact with bare hands, wear gloves
- Take maximum care when pouring off the supernatant in the falcon tube to prevent any splash and contamination
- Cover slips break easily, and if they are used and put in with the slides, they may break and cut the hands of the person washing them
- Clean the work surface after the work is finished with 1% bleach and 70% alcohol

# Sample

- Fresh faecal samples should be used in this procedure
- Process the faecal samples as soon as they arrived at the laboratory. If delay is unavoidable, process them within 24 hour of collection
- Do not refrigerate or freeze the faecal samples

# Materials, reagents and equipment’s

- Stool collection cup
- Petri dish (80ml in diameter)
- Tissue paper
- Wooden applicator stick
- Spatula
- Activated charcoal
- Funnel
- Rubber tube (35cm)
- Mohr clamp
- Strainer
- Haemostatic clamp
- Plastic Jar
- Laboratory tissue paper
- Test tube racks
- Timer
- Funnel stand( a wood plank in which hole shave been made to support several funnels)
- Scale
- stove
- Centrifuge
- Water bath (37 ^o^C)
- Plastic Pasteur pipettes
- Gloves
- Microscopic slide
- Coverslips
- Iodine
- Microscope
- Waste container
- Labels

# Procedure

**Step 1: Sample preparation**

- Using clean and labeled stool cup, weigh 10 g of fresh stool sample using scale (digital balance)
- Mix the stool sample with a very little amount of lukewarm water till the consistency becomes smooth (like peanut butter)
- Add 2 g activated charcoal and mix it till homogenization is made
- Label a petri dish and place 2 pieces of tissue paper on it
- Place the faecal material at the center of the tissue paper and fully cover the stool sample by single layer of tissue paper
- Close the petri dish and Incubate it at 26°C for 18 - 24hr

**NB:** The addition of very little water to the stool sample enables it to homogenize and make the stool sample smooth that can be spread easily in the petri dish.

**Step 2: Sample filtration**

- Turn on the water bath at 37^o^C
- Take the incubated stool sample out from the incubator
- Adjust 35cm rubber tube to the stem of a funnel
- Support the funnels in the funnel stand
- Close the rubber tubes with a Haemostatic clamp at the bottom and checked with lukewarm (37 ^o^C) water for any leakage
- Place a strainer over the funnel
- Place the stool sample over the strainer facing down the single layer tissue paper to the strainer
- Cover the stool sample with lukewarm water and leave it to stand for 1 hour at room temperature
- After an hour, lock the rubber tubes with artery forceps near the stem of the funnel
- Remove the haemostatic clamp and collect the water in 15 ml conical labeled 15 mL falcon tubes
- Centrifuged the tubes at 2000rpm for 5 minutes
- Decant the supernatant and leave the sediment (1 mL)

**Step 3: Microscopic examination**

- Check the sample identification
- Bring a droplet of the sediment using a Pasteur pipette to a microscope slide, do not add a cover slip
- Examine the slide under a microscope at low power (4x10 or 10 x 10)
- Only in case larvae are found, add a drop of iodine to kill the larvae, add a cover slip and examine further at low (10x10) or high (10x40) power
- Examine a new droplet and repeat this until the full sediment has been examined
- Record the result

**Remark**

- Read the slides systematically


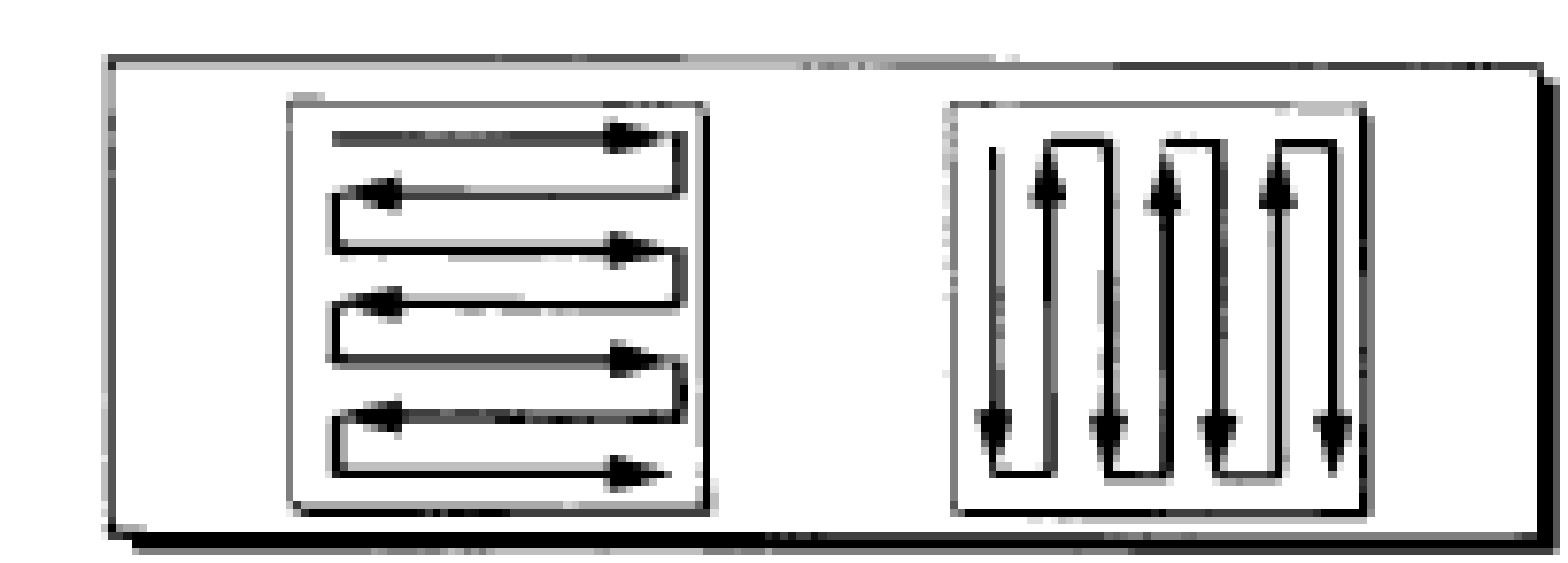


## Reporting of results

# Reporting of Results

- Use the ***“WHO Bench Aids in the Diagnosis of Intestinal Infections”*** or refer to color plates of parasites for morphological identification.
- Report any parasite seen

# Quality control

- Use clean, dry and leak proof containers for sample collection
- All larvae found should be confirmed with second microscopist to ensure whether it is *Strongyloides* or not
- The microscope should be checked for its functionality

# General remarks

## Waste management

- Testing materials should be disposed of in accordance with local, state and/or federal regulations.

## Precautions

You may get *Strongyloides* infection from splashes when discarding the supernatant. Therefore; you should handle samples and all residues especially after filtration.

# Reference

1. Aramendia AA, Anegagrie M, Zewdie D, Dacal E, Saugar JM, Herrador Z, et al. Epidemiology of intestinal helminthiases in a rural community of Ethiopia: Is it time to expand control programs to include Strongyloides stercoralis and the entire community? PLoS Negl Trop Dis. 2020; 14(6): e0008315. https://doi.org/10.1371/journal.pntd.0008315
2. World Health Organization. Bench aids for the diagnosis of intestinal parasites, second edition, WHO, Geneva. 2012.
