# Supplementary material for: Performance evaluation of Baermann techniques: The quest for developing a microscopy reference standard for the diagnosis of Strongyloides stercoralis
Source: PLoS Negl Trop Dis. 2021 Feb 18;15(2):e0009076. doi: 10.1371/journal.pntd.0009076 (PMC7891789; doi:10.1371/journal.pntd.0009076)
Supplement: S2 Text — (DOCX) [file pntd.0009076.s004.docx]

**Standard Operating Procedure for Modified Bearmann (MB)**

# Introduction

The Baermann technique is used to separate larvae from faecal material based on the active migration or movement of larvae downward from fresh stool samples to water of warmer temperature. Faeces are suspended in water. After permitting sufficient time to allow migration, the supernatant of the warm water will be discarded and the sediment will be examined microscopically for the presence of the larvae.

# Safety

- Stool samples should be treated as potentially infectious and universal precautions should be always followed
- Avoid contact with bare hands, wear gloves
- Take maximum care when pouring off the supernatant in the falcon tube to prevent any splash and contamination
- Cover slips break easily, and if they are used and put in with the slides, they may break and cut the hands of the person washing them
- Clean the work surface after the work is finished with 1% bleach and 70% alcohol

# Sample

- Fresh faecal samples should be used in this procedure
- Process the faecal samples as soon as they arrived at the laboratory. If delay is unavoidable, process them within 24 hour of collection
- Do not refrigerate or freeze the faecal samples

# Materials, reagents and equipment’s

- Stool collection cup
- Spatula
- Cotton wool gauze (8 layers – non-sterile, 100% cotton) of 5x5 cm)
- Wooden applicator stick
- 50 mL falcon tubes
- Microscopic slide
- Cover slip
- Timer
- Iodine
- Test tube racks
- Plastic Jar
- Laboratory tissue paper
- Labels
- Microscope
- Waste container
- Water bath (37 ^o^C)

# Procedure

**Step 1: Sample preparation**

- Fill a 50-ml tube with around 35 mL of lukewarm water (37 ^0^C)
- Place 2 pieces of cotton-wool gauze on top of each other in the Petri dish and bring 3 g of faeces on this napkin
- Make an opening on each corner of the napkins with a scissor or small knife
- Form a pouch containing the faecal material by putting a wooden stick through the 4 openings
- Place the pouch with the faecal material in the 50-ml tube; make sure the pouch only lightly touches the water but is not fully immersed
- Leave the 50-ml tube to stand for 2:30 hours at room temperature
- Decant the supernatant fluid and leave the sediment (3 mL) to settle for another 30 minutes

**Step 2: Microscopic examination**

- Check the sample identification
- Bring a droplet of the sediment using a Pasteur pipette to a microscope slide, do not add a cover slip
- Examine the slide under a microscope at low power (4x10 or 10 x 10)
- Only in case larvae are found, add a drop of iodine to kill the larvae, add a cover slip and examine further at low (10x10) or high (10x40) power
- Examine a new droplet and repeat this until the full sediment has been examined
- Record the result

**Remark**

- Read the slides systematically


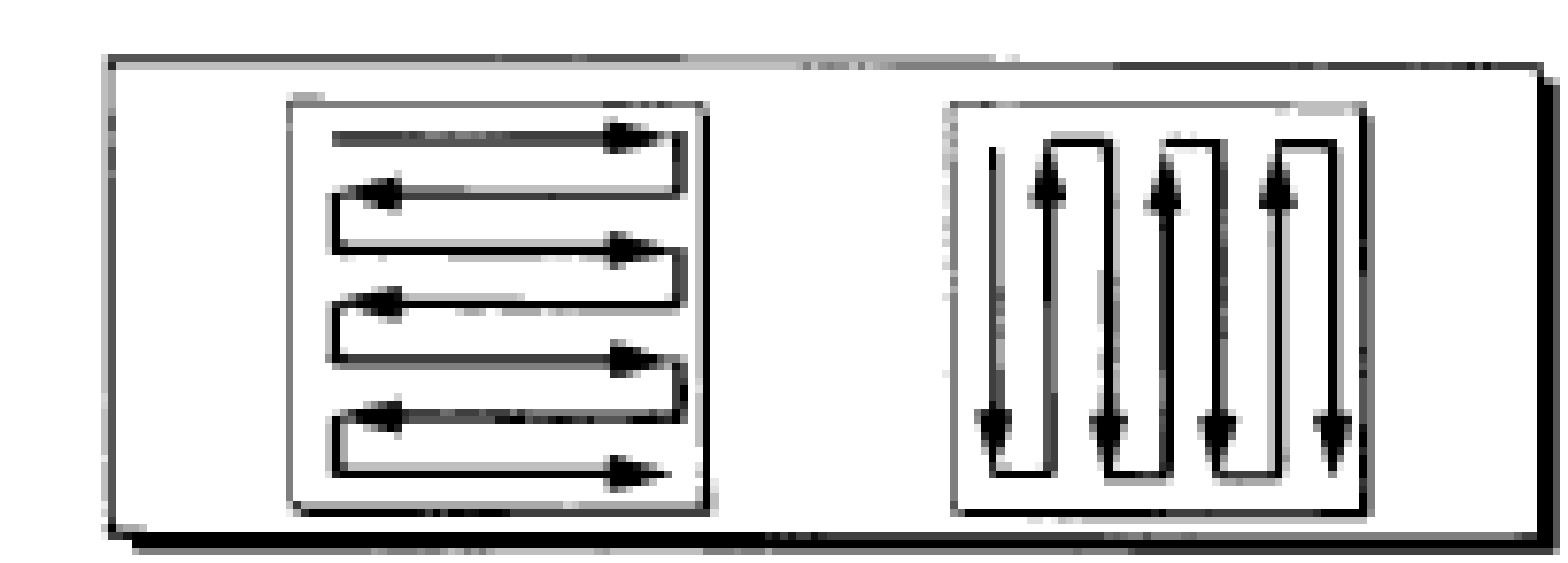


## Reporting of results

# Reporting of Results

- Use the ***“WHO Bench Aids in the Diagnosis of Intestinal Infections”*** or refer to color plates of parasites for morphological identification.
- Report any parasite seen

# Quality control

- Use clean, dry and leak proof containers for sample collection
- All larvae found should be confirmed with second microscopist to ensure whether it is *Strongyloides* or not
- The microscope should be checked for its functionality

# General remarks

## Waste management

- Testing materials should be disposed of in accordance with local, state and/or federal regulations.

# Reference

- 1. Meurs L, Polderman AM, Vinkeles NVS, Brienen EAT, Verweij JJ, Groosjohan B et al. Diagnosing Polyparasitism in a High-Prevalence Setting in Beira, Mozambique: Detection of Intestinal Parasites in Fecal Samples by Microscopy and Real-Time PCR. PLOS Neglected Trop Dis. 2017;11(1). http:// doi.org/10.1371/journal.pntd.0005310
  2. World Health Organization. Bench aids for the diagnosis of intestinal parasites, second edition, WHO, Geneva. 2012.
