# Supplementary material for: Performance evaluation of Baermann techniques: The quest for developing a microscopy reference standard for the diagnosis of Strongyloides stercoralis
Source: PLoS Negl Trop Dis. 2021 Feb 18;15(2):e0009076. doi: 10.1371/journal.pntd.0009076 (PMC7891789; doi:10.1371/journal.pntd.0009076)
Supplement: Attachment — Submitted filename: Rev Comments.docx [file pntd.0009076.s006.docx]

The study has omissions and mistakes in methodology which make its valuation difficult. Issues rising from methodology and discussion/conclusions are:

- Line 63: “Confirmed” ??
- Line 71: Reference 6: “Parasitic disease epidemiology” What is this reference? Please check “*Epidemiology* *of intestinal helminthiases in a rural community of Ethiopia: Is it time to expand control programs to include Strongyloides stercoralis and the entire community?* “ <https://doi.org/10.1371/journal.pntd.0008315>
- Line 87: formalin-ethyl acetate (or ether) concentration procedure. This is not a technique used for S. stercoralis detection an should not be mentioned as such.
- Line 143: ten grams of stool sample …..were, rather than was
- Line 142-144: the conventional Baermann technique does not include incubation with charcoal 18-26 h.
- Line 155-161: This is not a modified Baermann, but a conventional one.

The description of the three methods is confusing and wrong. In the first technique, you mention as conventional Baermann the one you describe with a pre-incubation with charcoal. The third one again has being done with charcoal pre-incubation. And the second one, the modified Baermann seems a conventional Baermann. I think you are comparing a conventional Baermann with 3 grams, with a modified Baermann with charcoal pre-incubation with 3 and ten grams and with a different proportion of charcoal (3/1, 10/6). Please revise the description. The last description is poor indeed (MBCI) and confusing. Did you use gauze or paper? Did you centrifuge?

Once you are using acronyms don’t use more the full word (e.g. Modified Baermann)

Could you be more precise about the centrifugation process?

Related to microscopy examination, the identification of larvae should be clarified. How did you identified *S. stercoralis*? Did you discarded hookworm co-infection, common in the area you are working in?

- Line 169-173: this reference standard test is weak, since you are comparing three modifications of the same technique. Please consider for limitations of this in the discussion.
- Lines 174-183: 174 “Time and cost required to perform each Baermann technique”: This explanation is not useful in the context of a scientific paper, as it is well known that Baermann technique is cheap and easy to perform.
- Line 239-241: “The lesser time required to analyze the samples by MBCI when compared to CB enables the technicians to handle more sample per person per day that will subsequently result in decreasing labor cost especially in a scenario” 2 min difference it’s not really a difference. The strongest of the technique in this case is in the results.
- Lin 251-255: this comment will be better in the introduction
- Line 265: I will advise to change reference 6 (*Epidemiology* *of intestinal helminthiases in a rural community of Ethiopia: Is it time to expand control programs to include Strongyloides stercoralis and the entire community?)*
- To assure that the Baermann technique is *“a useful method in the assessment of treatment response in clinical trials”* is incorrect: Firstly, there is a study , above mentioned, which was done in the same area you are working in and with the same modified Baermann with charcoal pre-incubation you used ( “*Epidemiology* *of intestinal helminthiases in a rural community of Ethiopia: Is it time to expand control programs to include Strongyloides stercoralis and the entire community*?”) with an overall prevalence of *S. stercoralis* of 56%, because of a combination of techniques was used. In your study you are not diagnosing all the infected population. In this line, the sensitivity of the Baermann technique when used as a single technique and when using one sample is not good, (particularly in chronic infections). As for treatment efficacy, Baermann will overestimate it. Even though a pre-incubation with charcoal increases the sensitivity of the technique, still is far from optimal.
- Limitations of the study should be discussed.
