# Supplementary material for: Performance evaluation of Baermann techniques: The quest for developing a microscopy reference standard for the diagnosis of Strongyloides stercoralis
Source: PLoS Negl Trop Dis. 2021 Feb 18;15(2):e0009076. doi: 10.1371/journal.pntd.0009076 (PMC7891789; doi:10.1371/journal.pntd.0009076)
Supplement: Attachment — Submitted filename: Response to reviewers.docx.docx [file pntd.0009076.s007.docx]

**Dear Editor and Reviewers,**

We thank the Reviewers for carefully evaluating our manuscript and for their constructive feedback. We acknowledge the comments, suggestions and questions raised. Please find below a point-by-point response (highlighted in yellow). We have revised the manuscript accordingly and all modifications are highlighted with track changes.

**Questions by reviewers**

***Reviewer #1:***

***Remarks to the Author:***

While advances are being made in the molecular diagnosis of *Strongyloides stercoralis*, this report makes is clear there is a need for an accurate and cheap standardized method for use in poor endemic areas. The authors therefore tested 3 modifications of the Baermann technique that are now in use to see which produced the most accurate diagnosis (the presence or absence of a living stage of S. stercoralis in the tested feces). The number of samples tested by each method was close to the calculated minimum needed. The statistics used were appropriate for this type of study. Ethical approval and regulatory approval were obtained before running the study.

The tables and figures are clear and the results are clearly presented. The study's analysis follows the plan to compare the 3 techniques on samples from the same study population. However the data produced by the study could have been analyzed further allowing further comparisons to published data.

The necessity of a cheap but accurate microscopy technique for the diagnosis of S. stercoralis in endemic areas (very often within very poor countries) is made clear in the introduction of this study. Without such a test the goal of implementing a strategy to eliminate *S. stercoralis* cannot begin. The authors' results also make it clear that one of the 3 techniques tested is significantly better that either or both of the other 2 and the cost is about the same as either of the inferior techniques.

This study concentrates on the cheap, accurate and widely used Baermann technique for the diagnosis of *S. stercoralis* while most recent publications compare expensive and hard to preform molecular techniques to microscopy techniques that are currently in place. Until the third world catches up to the standards needed to run PCR diagnostics (and recent events with CoVid-19 suggest even the developed world has a way to go!) only tests with few "moving parts" are going to be useful. As the authors state there must be a "gold standard" Baermann test for the diagnosis of S. stercoralis before we can move on to eliminating its threat. The authors could have used the Discussion to suggest the steps needed to run a gold standard Baermann.

RESPONSE: We are grateful for the reviewer’s valuable insight. We provided a response to all reviewers’ concerns below and addressed them in the manuscript.

1. Line 98: Do you mean the Baermann technique is usually reserved (“mostly”) for field studies and clinical trials, or that the technique is the most often technique used in field studies and clinical trials? Please re-word this sentence to make your meaning clear. Where does it stand among techniques currently used for clinical diagnosis?

We have clarified the language used. We meant to say that, among the microscopy-based techniques for the detection of *Strongyloides* larvae, the Bearmann technique is the technique most often used in field studies and clinical trials.

2. Lines 103-104: Why did you pick these three methods of running a Baermann technique for *S. stercoralis*, as I am sure that there are many other modifications used in diagnostic labs? Do you have any data on what proportion of clinical diagnostic labs are using each of the 3 methods you compare?

RESPONSE: The conventional Baermann method is a recommended technique for the diagnosis of *S. stercoralis* infections. It is by far the most often used technique in the field. Although sensitive, it is rather labour-intensive, time consuming, requires a lot of laboratory space, is cumbersome and the set-up requires large equipment and additionally large volumes of faeces. In the literature, several ways have been described to perform the Baermann method. However, our objective is to develop a simple, inexpensive yet sensitive microscopic reference standard for the diagnosis of *S.stercoralis* in large scale settings. We thus compared the conventional Baermann method (the most often used) to a simplified Baermann procedure (#4) from the Leiden University Medical Center’s (LUMC) in the Netherlands which didn’t employ pre-charcoal incubation versus a third method being the simplified adopted LUMC method with pre-charcoal incubation. In the latter two, the use of a funnel or a flask to deposit the faecal material from each sample is omitted for space saving and simplicity.

With regards to data on what proportions of clinical diagnostic labs are using each of the three methods we compared, we don’t have exact proportions of diagnostic labs which use the three methods. Our laboratory and others in the literature used different approaches of Baermann. For example:

**The modified Baermann** was used in this study, “Meurs L, Polderman AM, Vinkeles NVS, Brienen EAT, Verweij JJ, Groosjohan B et al. Diagnosing Polyparasitism in a High-Prevalence Setting in Beira, Mozambique: Detection of Intestinal Parasites in Fecal Samples by Microscopy and Real-Time PCR. PLOS Neglected Trop Dis. 2017;11(1). http:// doi.org/10.1371/journal.pntd.0005310”. **Conventional Baermann** tecnique and another **modification of Baermann technique** were used in this study, “Kiki-Barro PCM, Aka HJ, Kassi FK, Henriette VBH, Konaté A, Angora EK, et al. Comparative study between the Baermann conventional and a simplified Baermann device for the diagnosis of *Strongyloides stercoralis*. Afr. J. Parasitol. Res. 2017; 4 (5): 219-226”. On the other hand, in our laboratory **conventional Baermann** **with charcoal pre-incubation** method was used in the study of “Aramendia AA, Anegagrie M, Zewdie D, Dacal E, Saugar JM, Herrador Z, et al. Epidemiology of intestinal helminthiases in a rural community of Ethiopia: Is it time to expand control programs to include Strongyloides stercoralis and the entire community? PLoS Negl Trop Dis. 2020; 14(6): e0008315. https://doi.org/10.1371/journal.pntd.0008315” and “Hailu T, Nibret E, Amor A, Munshea A and Anegagrie A. Efficacy of single dose Ivermectin against *Strongyloides stercoralis* infection among primary school children in Amhara National Regional State. Infectious Diseases: Research and Treatment. 2020; 13:1–6’’

When we have noticed the lack of standardization of the Baermann procedures, we have conducted a pilot study that compares the **conventional Baermann** as described in “Kiki-Barro PCM, Aka HJ, Kassi FK, Henriette VBH, Konaté A, Angora EK, et al. Comparative study between the Baermann conventional and a simplified Baermann device for the diagnosis of *Strongyloides stercoralis*. Afr. J. Parasitol. Res. 2017; 4 (5): 219-226”, the **conventional Baermann with charcoal pre-incubation** with 10 g stool sample as described in reference #6 and the **modified Baermann** as described in reference #4. We choose this modified Baermann adopted from LUMC because it is simple, fast, needs simple tolos and did not take up much space.

We collected 70 stool samples from school children and processed with the tree methods.

From this pilot study, a total of 13 positive samples were detected by the three Baermann approaches. Out of 70 samples, only 2.9% (2/70) were positive using conventional Baermann and modified Baermann. 15.7% (11/70) samples were positive by conventional Baermann with charcoal pre-incubation technique. Only one sample was positive by all the three methods.

Since we thought the charcoal pre-incubations increases the positivity rate, we have modified again the **modified Baermann** to **modified Baermann with charcoal pre-incubation** using gauze as the procedure is described in our study. Then we have compared **CB (with charcoal pre-incubation)**, **MB** and **MBCI (the new approach that we have modified)**.

3. Lines 126-130: You calculated using Buderer’s formula that you needed a minimum of 432 samples for your study, but you only used 400 - 414 for any one test (364 by all 3 techniques). Why didn’t you collect enough samples to make your calculated minimum for each test run?

RESPONSE: We have revised the reference and the calculation. For the total sample size calculation, we used a prevalence of 32% according to reference # 6 as reported in a conference preceding. However, on publication of reference # 6, the prevalence was reported to be 33%. Thus, using 32% prevalence, the estimated sample size is 432. Using 33% prevalence, the estimated sample size is n = 420 (closer to the observed sample size of 400-414 or 364 by all 3 techniques. Fine tuning the calculation using the confidence intervals, the estimated prevalence of 33.0% (95 CI 30.0% - 36.3%) suggests a sample size of 461 and 386 respectively taking into account the lower and upper bounds. Thus, the calculated sample size for the comparison ranges from400 – 414 which covers the minimum required sample size.

4. Line 209-214: You give a breakdown of the sex and age of your participants, but you don’t bother to test if infection with *S. stercoralis* is significantly different in any subgroup or village. You should statistically test the results in each subgroup and report the significance if any. You could also let the reader know if any of the 3 techniques found significant number of infections within a subgroup while the other 2 did not.

RESPONSE: We thank the reviewer for pointing this out. We have done the analysis of Strongyloidiasis positivity and negativity in relation to socio demographic characteristics. Age (5-14), sex (female) and residence (being a resident in Gudguad) were found to be statistically significant as shown in Table 1. We didn’t find any significance difference between infections within the sub group and the three methods.

We have included this table in the manuscript with a detailed explanation on the significant results with P-value <0.05.

**Table 1: Strongyloidiasis Positivity and Negativity in relation to demographic characteristics**

| Socio demographic characteristics | | Positive  N (%) | Negative  N (%) | Total  N (%) | AOR  (95%CI) | P- Value |
| --- | --- | --- | --- | --- | --- | --- |
| Age | <5 | 1 (20) | 4 (80) | 5 (1.1) | 2.64 (.237 – 29.38) | .429 |
|  | 5 -14 | 42 (30) | 98 (70) | 140 (32) | 2.96 (1.38 – 6.34) | .005 |
|  | 15 – 49 | 87 (34.8 ) | 163 (65.2) | 250 (57.2) | 1.86 (.919 – 3.79) | .084 |
|  | >49 | 21 (50) | 21 (50) | 42 (9.6) | 1 | - |
| Sex | Female | 80 (29.9) | 188 (70.1) | 268 (61.3) | 1.69 (1.09 – 2.58) | .017 |
|  | Male | 71 (42) | 98 (58) | 169 (38.7) | 1 | - |
| residence | Baynesa | 23 (45.1) | 28 (54.9) | 51 (11.7) | .241 (.058 - .998) | .050 |
|  | Gedro | 5 (9.6) | 47 (90.4) | 52 (11.9) | 1.958 (.394 – 974) | .412 |
|  | Gudguad | 53 (47.3) | 59 (52.7) | 112 (25.6) | .213 (.055 - .828) | .026 |
|  | Kazimosh | 28 (40.6) | 41 (59.4) | 69 (15.8) | .292 (.072 – 1.178) | .084 |
|  | Sesaberet -1 | 22 (33.3) | 44 (66.7) | 66 (15.1) | .398 (.099 – 1.598) | .616 |
|  | Sesaberet -2 | 17 (25) | 51 (75) | 68 (15.6) | .694 (.167 – 2.88) | .616 |
|  | Zenzelima | 3 (15.5) | 16 (84.2) | 19 (4.3) | 1 | - |

5. Line 215: The MBCI modification most likely would have worked even better with more feces, was the size of the 50 ml tube’s opening the limiting factor (i.e. you used less feces to limit the size of the fecal pouch to that which would fit into the tube)?

RESPONSE: The 3 g stool sample used in the MBCI is in accordance with the LUMC SOP. The LUMC explored the option of using larger stool volumes in combination with larger containers, but this did not increase the diagnostic sensitivity (unpublished observation). Probably a large stool volume in the pouch, without any preincubation step, impedes the migration of the larvae to the water surface. More importantly, in the context of large scale screening and in clinical trial setting, the volume of collected stool sample tends to be an important limiting factor. Sufficient stool is needed in order to perform all assays to address varying endpoints. Factoring in polyparasitism alone suggests doing at least KK and Baermann techniques for all STH studies and stool quantities are not very abundant.

6. Line 243: Why did you make the assumption in determining the cost that you would compare 24 CB tests daily to 40 of the other 2 tests daily?

RESPONSE: Our assumption is based on an 8 hour working schedule per day and considering only the laboratory hands on time for each of the Baermann techniques. Based on this, we managed to process 24 samples using the CB and 40 samples using the MB or MBCI methods per day. Thus, we calculated the cost based on the number of samples which can be processed per day.

7. Lines 264-265: Your study found 34.6% infection rate (3 combined tests) compared to reference #6 rate of 33% by Baermann (method not described in this meeting abstract). The overall rate of infection found in reference #6 was 56% using the Baermann and 2 other tests. Do you know which of your 3 methods most closely corresponds to the Baermann method used in reference #6?

RESPONSE: In reference #6, the authors used a conventional Baermann with 30 g of stool sample. This procedure is similar to the one we used in our conventional Baermann method (CB), but we used 10 g of stool sample. On the other hand, in reference #6 the authors also used Formol-Ether Concentration (FEC) and PCR. Given the high sensitivity of PCR, it allows them to reach an infection rate of 56%.

8. Line 44: “that” should be “the”.

RESPONSE: We thank the reviewer for this grammar correction.

**Reviewer #2:**

**Remarks to the author:**

This manuscript reports on different variations of the Baermann technique for the microscopic diagnosis of Strongyloides stercoralis. Strongyloidiasis is increasingly being recognized as one of the clinically most important helminth infections, and standard diagnostic tools are insufficiently sensitive. The considerably varying reported diagnostic accuracies for the different detection techniques (Baermann, nutrient agar plate, PCR) might partially be explained by a lack of standardization of these techniques. Hence, the current manuscript is of importance and I support documentation of this work in published form.

RESPONSE: We are grateful for this feedback. Below you will find our response to the questions raised.

- Introduction & Methods: It would be helpful to provide a distinct reference for each of the three Baermann variations.

RESPONSE: We have followed the reviewer’s suggestion and reference the CB and MB method in the methods section. The MBCI method is our own modification of the MB to include charcoal pre-incubation to boost sensitivity.

- Methods: For the sample size calculation, you estimated a 32% prevalence, which is close to the actual prevalence found in the study. Did you have preliminary data on the occurrence of *S. stercoralis* in the study area or what assumption was this estimate based on?

RESPONSE: The sample size calculation was based on previous prevalence data from that area; “Aramendia AA, Anegagrie M, Zewdie D, Dacal E, Saugar JM, Herrador Z, et al. Epidemiology of intestinal helminthiases in a rural community of Ethiopia: Is it time to expand control programs to include Strongyloides stercoralis and the entire community? “PLoS Negl Trop Dis. 2020; 14(6): e0008315. https://doi.org/10.1371/journal.pntd.0008315. This reference is in the manuscript as reference # 6.

- Results: Please detail why five participants were excluded due to issues with the samples (too low quantity?).

RESPONSE: Five participants were excluded from the study because three of them couldn’t provide enough quantity of stool samples at the time of collection (they defecated and provided too low quantity). Two participants gave enough quantity of stool samples but contaminated with maggots, so we discarded these samples considering samples were not fresh.

- Results & Discussion: The authors show considerable differences in the prevalence obtained by the three modifications of the Baermann technique. Did they develop specific SOPs for the different protocols and could these be shared, e.g. as supplementary files? Alternatively, a schematic figure illustrating the three test modifications would be really useful. Additionally, it would be helpful to discuss e.g. the effects and mechanisms of charcoal incubation and the different gauze layers more thoroughly. What about other factors, e.g. type of water used, water temperature and the role of light, which is also commonly used to facilitate excretion of *S. stercoralis* larvae from the pouch to the Baermann tube?

RESPONSE: Yes, we have prepared SOPs for each of the procedures and attached them as supporting information (S3, S4, and S5). We used same type and layer of gauze in both MB and MBCI. Regarding the other factors, the stool samples were processed by the three techniques simultaneously using same the type of water, water temperature, and in same laboratory set up with similar lighting condition. So we didn’t consider them to affect the results between the three techniques.

- Discussion: Some of the Baermann modifications lead to a prolonged incubation so that it takes 24 hours or more until results become available. Hence, when these modifications are used, there is not much time difference as compared to charcoal culture or Koga nutrient agar plates. This could be mentioned/discussed.

RESPONSE: We used 18 -24 hour incubation in our study. The copro-culture takes up to 7 days as described in “Meurs L, Polderman AM, Vinkeles NVS, Brienen EAT, Verweij JJ, Groosjohan B et al. Diagnosing Polyparasitism in a High-Prevalence Setting in Beira, Mozambique: Detection of Intestinal Parasites in Fecal Samples by Microscopy and Real-Time PCR. PLOS Neglected Trop Dis. 2017;11(1). http:// doi.org/10.1371/journal.pntd.0005310”. And the Koga nutrient agar plates needs 2 days (48 hours) as described in “Hailu T, Nibret E, Amor A, Munshea A and Anegagrie A. Efficacy of single dose Ivermectin against *Strongyloides stercoralis* infection among primary school children in Amhara National Regional State. Infectious Diseases: Research and Treatment. 2020; 13:1–6’’. Therefore; MBCI requires less time when compared to both methods.

- Discussion: I miss a part on limitations of this study. With all the detailed efforts made to accurately compare the different Baermann tests, would it not have been a good opportunity to perform also PCR and or Koga agar plate, in particular to obtain a more independent composite reference standard?

RESPONSE: We thank the reviewer for pointing out it. We have included it as limitation in the manuscript.

**Reviewer #3:**

**Remarks to the author:**

The objectives of the study are clear. Is the study design is appropriate and the sample size sufficient statistical analyses used are correct. Methodology description should be revised and clarified. The study has omissions and mistakes in methodology which make its valuation difficult.

Study of some interest in the field work with an important background in the main issue, which is the appropriate management of STH and the inclusion of *S. stercorlais* in the NTD.

**Response:** We are grateful for the feedback and have revised the methods section in the manuscript. Below is a response to the specific points raised.

- Line 63: “Confirmed”??

RESPONSE: A high prevalence of *S. stercoralis* infection has been reported before in our study area. To reduce possible confusion we replaced “confirmed” with “found”.

- Line 71: Reference 6: “Parasitic disease epidemiology” What is this reference? Please check “*Epidemiology* *of intestinal helminthiases in a rural community of Ethiopia: Is it time to expand control programs to include Strongyloides stercoralis and the entire community?* “ https://doi.org/10.1371/journal.pntd.0008315

RESPONSE: When we initially prepared our protocol, these finding have been published in a conference proceedings. Now this is published. We thank the reviewer for the suggestion and we have changed the reference.

- Line 87: formalin-ethyl acetate (or ether) concentration procedure. This is not a technique used for *S. stercoralis* detection and should not be mentioned as such.

RESPONSE: We agree with the reviewer and are grateful for bringing our attention to this point. We have removed it and rephrased it as “Commonly used techniques are the Baermann technique and stool culture, for which either plain agar plates or a charcoal copro-culture procedure can be used. Stool cultures have a relatively high sensitivity, since they allow the parasite to enter the free living cycle, but the procedure is cumbersome and time-consuming (13)”.

- Line 143: ten grams of stool sample …..were, rather than was

RESPONSE: We thank the reviewer for the grammar correction. We have modified the manuscript accordingly to “Ten grams of stool sample were weighed and mixed with two grams of activated charcoal and lukewarm water.”

- Line 142-144: the conventional Baermann technique does not include incubation with charcoal 18-26 h.

RESPONSE: Kindly, find our response below.

- Line 155-161: This is not a modified Baermann, but a conventional one.

The description of the three methods is confusing and wrong. In the first technique, you mention as conventional Baermann the one you describe with a pre-incubation with charcoal. The third one again has being done with charcoal pre-incubation. And the second one, the modified Baermann seems a conventional Baermann. I think you are comparing a conventional Baermann with 3 grams, with a modified Baermann with charcoal pre-incubation with 3 and ten grams and with a different proportion of charcoal (3/1, 10/6). Please revise the description. The last description is poor indeed (MBCI) and confusing. Did you use gauze or paper? Did you centrifuge?

RESPONSE: We appreciate this comment and the comment above, as they, together with the comments of the other reviewers, elegantly illustrate that there is no generally accepted standardized procedure and terminology concerning the microscopic detection of *Strongyloides* larvae. Around the world slightly different techniques are used and the definition of what is considered as “conventional” might be contradicting. We considered the Baermann technique as described in the manuscript as conventional and modified it based on the materials and equipment used in each procedures. For further clarification we included again the references for CB and MB in the paragraphs describing the followed procedures as well as in the supplementary information.

- Could you be more precise about the centrifugation process?

RESPONSE: For the CB method, we used centrifugation at 2000 rpm for 5 minutes and this is included in the manuscript.

- Related to microscopy examination, the identification of larvae should be clarified. How did you identify *S. stercoralis*? Did you discard hookworm co-infection, common in the area you are working in?

RESPONSE: We didn’t discard co-infections rather used morphological identifications criteria for all positive samples to differentiate *S. stercoralis* from hookworm species.

For rhabditiform larvae; the length of buccal cavity (short) and esophagus (one-third), prominent genital permordium was considered while for filariform larvae; the length of esophagus (half of the body), notched tail and absence of sheath was considered.

- Line 169-173: this reference standard test is weak, since you are comparing three modifications of the same technique. Please consider for limitations of this in the discussion.

RESPONSE: We have included the following paragraph in the discussion, describing the fact that we could not use PCR or Koga. “The fact we used a single stool sample might, despite an optimal Baermann procedure, still give an underestimation of the actual prevalence of *S. stercoralis* infection in our study population. Alternative techniques with a higher sensitivity, such as Koga agar plate culture or PCR, could not be used due to the lack of appropriate laboratory facilities. In general, techniques based on the detection of parasite DNA remain a challenge in many regions where strongyloidiasis is endemic”.

- Lines 174-183: 174 “Time and cost required to perform each Baermann technique”: This explanation is not useful in the context of a scientific paper, as it is well known that Baermann technique is cheap and easy to perform.

RESPONSE: Even though Baermann technique is cheaper when compared to other techniques, our objective was to select the most simple, sensitive, and low cost diagnostic technique among the different Baermann techniques, as this is of relevance especially for large scale screening.

- Line 239-241: “The lesser time required to analyze the samples by MBCI when compared to CB enables the technicians to handle more sample per person per day that will subsequently result in decreasing labor cost especially in a scenario” 2 min difference it’s not really a difference. The strongest of the technique in this case is in the results.

RESPONSE: We agree with the reviewer that 2 minutes is a small difference but in a large scale screening initiative, 2 minutes per samples could have an impact. Our target is a diagnostic method for use in large scale screening.

- Lin 251-255: this comment will be better in the introduction

RESPONSE- We thank the reviewer for the suggestion.

- Line 265: I will advise to change reference 6 (*Epidemiology* *of intestinal helminthiases in a rural community of Ethiopia: Is it time to expand control programs to include Strongyloides stercoralis and the entire community?)*

RESPONSE: It is changed as per your suggestion.

- To assure that the Baermann technique is *“a useful method in the assessment of treatment response in clinical trials”* is incorrect: Firstly, there is a study , above mentioned, which was done in the same area you are working in and with the same modified Baermann with charcoal pre-incubation you used ( “*Epidemiology* *of intestinal helminthiases in a rural community of Ethiopia: Is it time to expand control programs to include Strongyloides stercoralis and the entire community*?”) with an overall prevalence of *S. stercoralis* of 56%, because of a combination of techniques was used. In your study you are not diagnosing all the infected population. In this line, the sensitivity of the Baermann technique when used as a single technique and when using one sample is not good, (particularly in chronic infections). As for treatment efficacy, Baermann will overestimate it. Even though a pre-incubation with charcoal increases the sensitivity of the technique, still is far from optimal.

Limitations of the study should be discussed.

RESPONSE: We agree with the reviewer and we have expanded on this in the manuscript.

The Baermann technique for the assessment of treatment response is challenging since lack of detection of larvae cannot always be indicative of cure. This is because of the individual patterns of larval shedding in stool. This limitation can be overcome by performing consecutive stool samples at screening and follow-up post treatment. The added benefit of the MBCI is that since it is simple to perform it can be employed for consecutive day screening. Although serology has shown a good performance as a test of cure in non-endemic settings (23), that tool is of little value in endemic areas where re-infection is likely.

Another drawback of the Baermann method is that, if stool samples contain hookworm eggs, these may hatch during the Baermann procedure. However this limitation can be overcome by distinguishing the morphology of the different species.

As described above, we added a small paragraph describing the limitation of a single stool sample examination and the fact that there are more sensitive procedures such as Koga agar plate culture or PCR, but there are not easy accessible in any endemic regions.
